# Association between metabolically healthy central obesity in women and levels of soluble receptor for advanced glycation end products, soluble vascular adhesion protein-1, and activity of semicarbazide-sensitive amine oxidase

**DOI:** 10.3325/cmj.2017.58.106

**Published:** 2017-04

**Authors:** Ivana Koborová, Radana Gurecká, Melinda Csongová, Katarína Volkovová, Éva Szökő, Tamás Tábi, Katarína Šebeková

**Affiliations:** 1 Institute of Molecular Biomedicine, Faculty of Medicine, Comenius University, Bratislava, Slovakia; 2 Institute of Medical Physics, Biophysics, Informatics and Telemedicine, Faculty of Medicine, Comenius University, Bratislava, Slovakia; 3 Institute of Biology, Faculty of Medicine, Slovak Medical University, Bratislava, Slovakia; 4 Department of Pharmacodynamics, Faculty of Pharmacy, Semmelweis University, Budapest, Hungary

## Abstract

**Aim:**

To determine the levels of circulating soluble receptor for advanced glycation end products (sRAGE), as a biomarker of risk of metabolic syndrome and cardiovascular disease development in centrally obese (CO) women considered metabolically healthy (COH) in comparison with those metabolically unhealthy (COU).

**Methods:**

47 lean healthy, 17 COH (presenting waist-to-height ratio ≥0.5 but not elevated blood pressure, atherogenic lipid profile, and insulin resistance), and 50 COU (CO presenting ≥2 risk factors) women aged 40-45 years were included. Anthropometric characteristics, blood chemistry and hematology data, adipokines, markers of inflammation, sRAGE, soluble vascular adhesion protein-1 (sVAP-1), and the activity of semicarbazide sensitive amine oxidase (SSAO) were determined.

**Results:**

Central obesity associated with low sRAGE levels (lean healthy: 1503 ± 633 pg/mL; COH: 1103 ± 339 pg/mL, *P* < 0.05; COU: 1106 ± 367 ng/mL, *P* < 0.0.1), hyperleptinemia, and elevated markers of inflammation irrespective of the presence or absence of cardiometabolic risk factors. COU women presented high adiponectin levels. SVAP-1 concentrations and the activity of SSAO were similar in all 3 groups.

**Conclusion:**

COH women present abnormalities in non-standard markers of cardiometabolic risk (sRAGE, leptin, high sensitive C-reactive protein), supporting the view that there is no healthy pattern of obesity. The clinical impact of our findings for future prognosis of metabolically healthy obese subjects remains to be elucidated in longitudinal studies.

Advanced glycation end products (AGEs) are adducts of reducing sugars or reactive aldehydes to proteins, lipoproteins or DNA. AGEs are formed *in vivo* spontaneously and mainly non-enzymatically in tissues and body fluids. Their excessive accumulation exerts negative health effects through modification of the structure and function of proteins or interaction with their specific cell surface receptor for AGEs – RAGE (1). AGE/RAGE interaction activates the nuclear factor kappa-B and other downstream pathways, resulting in production of reactive oxygen species and pro-diabetic, pro-inflammatory, pro-thrombotic, and pro-atherogenic responses (1). Circulating soluble RAGEs (sRAGE) comprise the endogenous secretory RAGE (esRAGE, an alternatively spliced variant of RAGE) and a proteolytically (by a disintegrin and metalloproteinase-10 [ADAM10] and a matrix metalloproteinase-9 [MMP-9]) cleaved form of the cell surface receptor (2,3). sRAGE levels are increased in chronic renal disease and diabetes, but low level of circulating sRAGE is considered a reliable biomarker for other diseases, such as metabolic syndrome and its components, atherosclerosis, coronary heart disease, and other conditions (4-6).

While majority of studies in subjects without diabetes reported an inverse relationship between sRAGE and measures of adiposity (4,6-9), other studies revealed no significant association (10,11). We speculated whether this discrepancy in findings might be explained by obese metabolically healthy phenotype, ie, obese subjects without increased blood pressure, atherogenic lipid profile, and insulin resistance (12). We investigated whether centrally obese metabolically healthy women presented higher sRAGE levels than their centrally obese metabolically unhealthy counterparts. We also evaluated the levels of soluble vascular adhesion protein-1 (sVAP-1) and its semicarbazide-sensitive amino oxidase (SSAO) activity. Both, RAGE and VAP-1 are cleaved from the cell surface by MMP-9 (3,13). SSAO converts less toxic primary amines into toxic reactive aldehydes – precursors of AGEs (14). Methylglyoxal-derived AGEs, such as hydroimidazolones and N^ϵ^-(carboxyethyl)lysine, are RAGE ligands (15). Thus, we anticipated that there might be a functional link between sRAGE and SSAO/sVAP-1.

## Subjects and methods

This cross-sectional study was conducted in accordance to the principles of the Declaration of Helsinki. All subjects signed an informed consent to participate.

### Subjects

Women were recruited via general practitioners or advertisements posted in busy locations. Inclusion criteria were age between 40 and 45 years and stable physical condition. Exclusion criteria were any acute and chronic illness, particularly diabetes mellitus, cardiovascular diseases or cancer, unstable physical condition, disabilities, consumption of >50 units of alcohol/week, any body weight lowering regimen, pregnancy, and breast-feeding.

Of 190 recruited women, 6 were initially excluded for fasting plasma glucose (FPG)>6.9 mmol/L (n = 4) and incomplete data for unequivocal determination of risk factors (n = 2). Of the remaining 184 women classified for presence of cardiometabolic risk factors, 88 were lean, ie, presented waist-to-height ratio (WHtR)<0.5. Forty-seven of them were lean healthy (LH), and 41 presented ≥1 risk factors, thus they were not included into the analysis. Ninety-six women were centrally obese. Seventeen were centrally obese healthy (COH), and 50 women were obese unhealthy women (COU). The remaining 29 centrally obese women presented only 1 additional risk factor, thus they were not included into the analysis. The remaining 184 women were categorized according to the presence of four cardiometabolic risk factors including central obesity, increased blood pressure (BP), increased atherogenic risk, and insulin resistance. Central obesity was defined as WHtR≥0.5 (16); increased blood pressure as systolic BP≥130 mm Hg and/or diastolic BP≥85 mm Hg; increased atherogenic risk as atherogenic index of plasma ((AIP) = log(triacylglycerols/high-density lipoprotein cholesterol))≥0.11 (17); and insulin resistance as quantitative insulin sensitivity check index (QUICKI = 1/(log_10_(18.82xfasting plasma glucose)+log_10_ fasting plasma insulin))≤0.320 (corresponding to 25th percentile in the cohort). Thereafter, 3 groups were selected for this analysis as follows: LH women without any of the four risk factors (n = 47); COH women with no other risk factor except central obesity (n = 17); and COU women presenting with additional ≥2 risk factors (n = 50). None of the included women was treated with antihypertensive and/or lipid lowering drugs.

### Methods

Body weight was measured using calibrated electronic balance, height using extendable stadiometer, and waist circumference using flexible inelastic belt-type tape. BP and heart rate were measured using digital sphygmomanometer at forearm in sitting position after 10-minute rest, and the mean value of the last 2 out of 3 measurements was recorded. Measurements were performed in the morning hours at the outpatient department.

Blood was taken from antecubital vein after overnight fasting. Standard blood chemistry (FPG, lipid profile, creatinine, uric acid, albumin; Vitros 250 analyzer, Johnson&Johnson, Rochester, NY, USA) and blood count (Sysmex K-20 analyzer, TOA Medical Electronics, Kobe, Japan) were analyzed immediately. Plasma samples were stored at -80°C for special analyses. Fasting plasma insulin (FPI) concentration was analyzed using a commercial radioimmunoassay (Immunotech, Prague, Czech Republic). Commercial ELISA sets were used according to manufacturers’ instructions to determine high sensitive C-reactive protein (hsCRP, Immundiagnostik AG, Bensheim, Germany), sVAP-1 (eBioscience, Vienna Austria), adiponectin, leptin, and sRAGE (all R&D Systems, Minneapolis, MN, USA). SRAGE ELISA assay used in our study determines all circulating sRAGE splice variants. Advanced glycation end products-associated fluorescence of plasma (AGE-Fl) and advanced oxidation protein products (AOPPs) were determined as described previously (10). SSAO activity in plasma was determined radiometrically by liquid scintillation counting as described previously (18). The enzyme activity was expressed as nmol of benzaldehyde formed by 1 mg plasma protein in 1 h at 37°C (nmol mg^-1^ h^-1^).

Body mass index (BMI), waist-to-height ratio, quantitative insulin sensitivity check index, atherogenic index of plasma, and glomerular filtration rate (eGFR; according to (19)) were calculated. AGE-associated fluorescence of plasma and AOPPs were expressed as their ratio to plasma albumin (Alb).

### Statistical analysis

Normality of data distribution and equality of variances were tested with Kolmogorov-Smirnov and Levene’s test, respectively. Data fitting to normal distribution are given as mean ± standard deviation (SD) and were compared using one-way analysis of variance (ANOVA) with post-hoc Scheffe’s test with correction of significance level for multiple comparisons. Skewed data are given as median and interquartile range. These groups were compared using Kruskal-Wallis test with subsequent Dunn’s post-hoc test with correction of significance level for multiple comparisons. Spearman or Pearson correlation coefficients (as appropriate) between sRAGE, sVAP-1 or SSAO activity and anthropometric, clinical, and laboratory variables were calculated. To uncover the independent variables with class discriminating ability, an orthogonal partial least squares discriminant analysis (OPLS-DA) was performed using Simca v.13 software (Umea, Sweden). Age, height, heart rate, eGFR, white blood cell (WBC), red blood cell (RBC), and platelet counts, albumin, uric acid, hsCRP, adiponectin, leptin, sRAGE and sVAP-1 concentrations, SSAO activity, AGE/Alb, and AOPP/Alb levels were entered as predictors. Variable of importance for the projection (VIP) value ≥1 was considered significant.

To assess which independent variables were significant predictors to selected dependent variables, a multivariate regression was performed using the general linear model (GLM). For sRAGE as dependent variable, those independent variables were entered as covariates, which showed significant association to sRAGE in simple regression analyses, ie, WHtR, SBP, DBP, QUICKI, AIP, eGFR, uric acid, ln hsCRP, ln adiponectin, ln leptin, AGE/Alb, ln AOPP/Alb, platelet count, SSAO activity, and sVAP-1. WHtR, ln hsCRP, and sRAGE were entered as independent predictors of SSAO activity. *P* < 0.05 was considered significant. Statistical analyses were performed using SPSS version 22 (SPSS Inc., Chicago, IL, USA) and GraphPad Prism v. 6.0 (California, USA).

The statistical power of the study was determined using the post-hoc power test. With 2-sided confidence interval of 95%, the power of comparison between 47 lean healthy subjects presenting mean sRAGE concentration of 1503 pg/mL with SD of 633 pg/mL and 17 COH subjects with sRAGE levels of 1103 ± 339 pg/mL was 89.9%.

## Results

COU women were significantly older in comparison with their lean healthy counterparts (Table 1), however, the age-difference of 9.6 months in mean cannot be considered in 40-year-olds as clinically significant. Both groups of centrally obese women tended to be shorter in comparison with lean healthy subjects, however, *post-hoc* Scheffe’s test failed to identify significant between group differences. In comparison with lean healthy subjects, COH women presented with significantly higher FPG, and lower insulin sensitivity (within the insulin sensitive range, ie, QUICKI≥0.320). COH and COU groups has similar characteristics of general and central obesity.

**Table 1:** Anthropometric characteristics, blood chemistry, and hematology data of women included in the study

	LH (n = 47)	COH (n = 17)	COU (n = 50)	P
Age (years)	42.0 (40.0; 43.0)	42.0 (41.5; 43.0)	42.0 (41.0; 44.0)*	0.015
Height (cm)	168.1 ± 6.9	163.4 ± 6.6	165.3 ± 7.7	0.037^†^
Weight (kg)	63.2 ± 8.0	82.3 ± 16.8^§^	89.4 ± 17.4^§^	<0.001
Waist (cm)	74.3 ± 6.2	94.1 ± 11.9^§^	97.7 ± 9.2^§^	<0.001
BMI (kg/m^2^)	22.3 ± 2.3	30.7 ± 5.3^§^	32.5 ± 4.7^§^	<0.001
Waist/height	0.44 ± 0.03	0.58 ± 0.07^§^	0.59 ± 0.05^§^	<0.001
SBP (mmHg)	116 ± 7	120 ± 6	139 ± 13^§,¶^	<0.001
DBP (mmHg)	74 ± 5	77 ± 5	92 ± 8^§,¶^	<0.001
Heart rate (x/min)	71 ± 9	72 ± 10	80 ± 12^§,+^	<0.001
FPG (mmol/L)	5.0 ± 0.4	5.3 ± 0.4*	5.6 ± 0.5^§^	<0.001
FPI (μIU/ml)	6.3 (5.5; 8.5)	8.1 (6.8; 10.6)	14.8 (10.2; 18.3)^§,¶^	<0.001
QUICKI	0.361 ± 0.020	0.344 ± 0.014*	0.318 ± 0.020^§,¶^	<0.001
Albumin (g/L)	45 ± 3	45 ± 5	44 ± 4	0.619
Cholesterol (mmol/L)	4.9 ± 0.9	5.5 ± 0.9*	5.4 ± 1.0	0.028^†^
HDL-C (mmol/L)	1.5 (1.4; 1.9)	1.5 (1.4; 1.8)	1.2 (1.0; 1.4)^§,¶^	<0.001
LDL-C (mmol/L)	2.9 ± 0.7	3.4 ± 0.8	3.3 ± 0.9*	0.014
VLDL-C (mmol/L)	0.4 (0.3; 0.5)	0.5 (0.4; 0.6)	0.8 (0.6; 1.0)^§,¶^	<0.001
TAG (mmol/L)	0.8 (0.7; 1.1)	1.1 (0.9; 1.3)	1.8 (1.3; 2.3)^§,¶^	<0.001
AIP	-0.29 ± 0.18	-0.17 ± 0.15	0.18 ± 0.23^§,¶^	<0.001
Uric acid (mmol/L)	211 ± 53	244 ± 63	280 ± 62^§^	<0.001
Creatinine (μmol/L)	73 ± 15	70 ± 9	70 ± 12	0.622
eGFR (ml s^-1^ (1.73m^2^)^-1^)	1.6 ± 0.4	1.6 ± 0.2	1.6 ± 0.3	0.798
WBC (x*10^9^/L)	6.2 ± 1.4	6.6 ± 1.4	7.7 ± 2.1^§^	<0.001
RBC (x*10^12^/L)	4.5 ± 0.4	4.5 ± 0.3	4.7 ± 0.4*	0.013
Plt (x*10^9^/L)	241 ± 67	258 ± 65	297 ± 64^§^	<0.001
hsCRP (mg/L)	3.1 (2.0; 4.1)	4.5 (3.3; 8.1)^‡^	5.7 (4.2; 9.2)^§^	<0.001
Adiponectin (ng/mL)	14.5 (9.8; 20.4)	12.8 (9.7; 18.6)	8.6 (5.6; 10.3)^§,‖^	<0.001
Leptin (ng/mL)	11.4 (6.4; 19.3)	38.5 (18.0; 66.6)^§^	44.0 (28.0; 57.0)^§^	<0.001
AOPP/Alb	1.2 (1.0; 1.4)	1.1 (0.9; 1.9)	2.7 (1.7; 4.4)^§,¶^	<0.001
AGE-Fl/Alb	55.4 ± 14.0	55.6 ± 15.3	52.3 ± 14.8	0.531

Presence of cardiometabolic risk factors was classified as follows: central obesity: WHtR≥0.5; elevated BP: systolic BP (SBP)≥130 mm Hg and/or diastolic BP (DBP)≥□85 mm Hg; increased atherogenic risk: AIP = log(TAG/HDL)≥0.11 (17); insulin resistance: QUICKI≤0.320 (corresponding to 25th percentile in the cohort) . Lean healthy (LH) women did not present any risk factor; centrally obese healthy (COH) women did not present any other risk factor except for central obesity; centrally obese unhealthy (COU) women presented ≥2 additional risk factors except for central obesity; BMI: body mass index; SBP: systolic blood pressure; DBP: diastolic blood pressure; FPG: fasting plasma glucose; FPI: fasting plasma insulin; QUICKI: quantitative insulin sensitivity check index; HDL-C: high density lipoprotein cholesterol; LDL-C: low density lipoprotein cholesterol; VLDL-C: very low density lipoprotein cholesterol; TAG: triacylglycerols; AIP: atherogenic index of plasma; eGFR: estimated glomerular filtration rate (abbreviated MDRD formula); WBC: leukocyte count; RBC: erythrocyte count; Plt: platelet count; hsCRP: high sensitive C-reactive protein; AOPP/Alb: advanced oxidation protein products corrected for plasma albumin; AGE-Fl/Alb: advanced glycation end products-associated fluorescence of plasma corrected for plasma albumin; AU: arbitrary units; data presented as mean ± SD or as median and interquartile range; normally distributed data were compared using one-way analysis of variance with Scheffe’s post-hoc test with correction for multiple comparisons; skewed data were compared using Kruskal-Wallis test with post-hoc Dunnet’s test with correction for multiple comparisons.

*P > 0.05 vs lean healthy.

†Scheffe’s test did not indicate significance between the groups

‡*P* > 0.01 vs lean healthy.

§*P* > 0.001 vs lean healthy.

‖*P* > 0.01 vs centrally obese healthy.

¶*P* > 0.001 vs centrally obese healthy.

### Standard blood chemistry and red blood cells and platelets counts

No significant differences in albuminemia were observed between the groups. COU women presented mildly elevated uric acid concentrations, significantly higher in comparison with lean healthy subjects. Markers of renal function (eg, plasma creatinine concentration and eGFR) did not differ significantly between the groups (Table 1). COU women had higher red blood cells and platelets counts in comparison with lean healthy subjects.

### Inflammatory markers

Both groups of centrally obese women presented higher hsCRP concentrations in comparison with their lean healthy counterparts. COU women had higher WBC counts in comparison with COH and lean healthy women (Table 1).

### Adipokines

COU women presented lower adiponectin concentrations in comparison lean healthy and COH subjects, while leptin concentrations were similarly increased in both groups of centrally obese women if compared with those lean and healthy (Table 1).

### Advanced glycation end products and advanced oxidation protein products

AGE-Fl/Alb levels did not differ significantly between the groups. COU women presented higher AOPP/Alb levels in comparison with lean healthy and COH groups (Table 1).

### sRAGE, sVAP-1 concentration and SSAO activity

**Figure 1:**
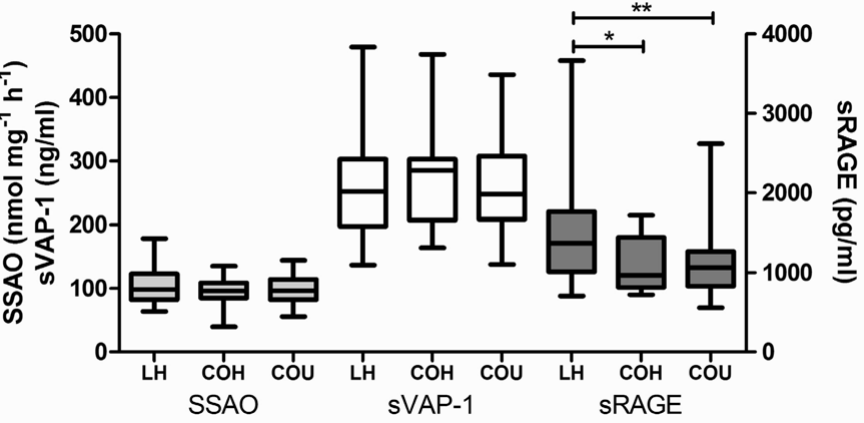
Activity of semicarbazide-sensitive amine oxidase, plasma soluble vascular adhesion protein-1 and soluble receptor for advanced glycation end products levels in lean healthy, centrally obese healthy and centrally obese unhealthy women. SSAO: activity of semicarbazide-sensitive amine oxidase; sVAP-1: soluble vascular adhesion protein-1; sRAGE: soluble receptor for advanced glycation end products; LH: lean healthy; COH: centrally obese healthy; COU: centrally obese unhealthy women. Data presented as minimum, first quartile, median, third quartile and maximum. Kruskal-Wallis test with subsequent Dunn's test with correction of P for multiple comparisons were used for statistical comparison. Significant differences between the groups are shown. *P < 0.05; **P < 0.01.

Both groups of centrally obese women presented lower sRAGE concentrations in comparison with their lean healthy counterparts (LH: 1370 (1011; 1765) pg/mL; COH: 969 (815; 1442) pg/mL; COU: 1062 (832; 1266) pg/mL; *P* < 0.001). (Figure 1). SVAP-1 concentration (LH: 252 (198; 303) ng/mL; COH: 285 (207; 303) ng/mL; COU: 248 (209; 309) ng/mL; *P* = 0.596) and SSAO activity (LH: 99 (82; 123) nmol mg^-1^ h^-1^; COH: 96 (85; 108) nmol mg^-1^ h^-1^; COU: 97 (82; 114) nmol mg^-1^ h^-1^; *P* = 0.627) did not differ significantly between the groups (Figure 1).

### Multivariate analysis.

**Figure 2:**
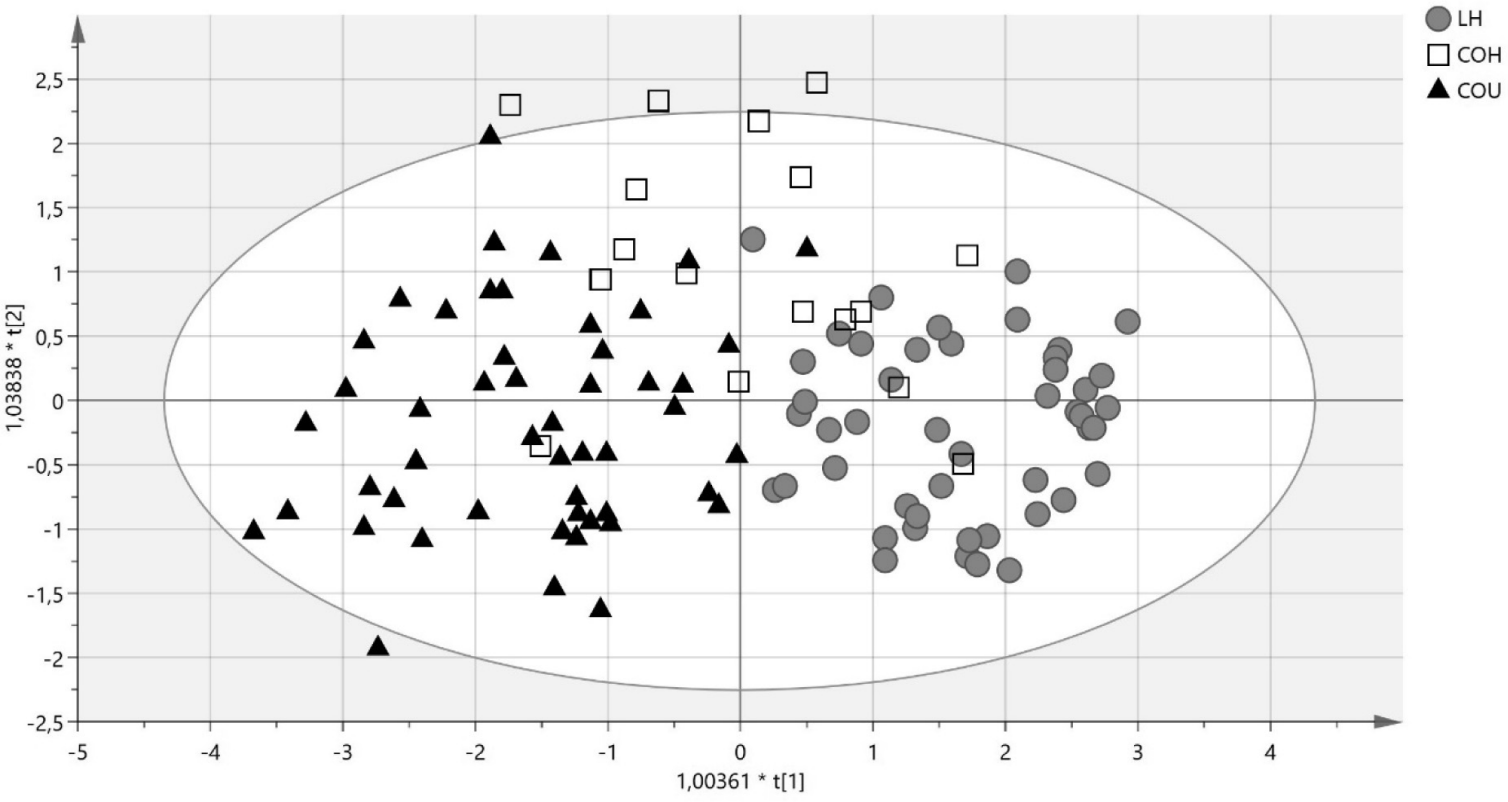
Score scatter plot from OPLS-DA model of lean healthy women, centrally obese healthy, and centrally obese unhealthy women. LH: lean healthy women, (gray circles); COH: centrally obese healthy women, (squares); COU: centrally obese unhealthy women, black triangles). Scores are orthogonal ( = completely independent from each other), representing new variables summarizing the input of all determined variables (herein age, height, heart rate, eGFR, WBC, RBC and platelet counts, albumin, uric acid, hsCRP, adiponectin, leptin, sRAGE and sVAP-1 concentrations, SSAO activity, AGE/Alb and AOPP/Alb levels) so that one score vector corresponds to one subject, having its own score vector. Observations situated outside Hotelling’s T2 tolerance ellipse are mild outliers. Model reveals that COH women scatter between the LH and COH women, while the two latter groups are well separated (separation in direction of x-axis). Separation in direction of y-axis represents within group variability.

OPLS-DA confirmed that COH women presented intermediate phenotype: COH subjects projected scattered among relatively well separated groups of lean healthy and COU women (Figure 2). Model explained 53% of between-groups variation. Leptin, hsCRP, WBC count, UA, sRAGE, adiponectin, sVAP-1 and heart rate were selected as independent predictors contributing significantly to separation between the groups (VIP values 1.53-to-1.00), (loading scatter plot and VIP plot given in supplementary material).

### sRAGE

**Figure 3:**
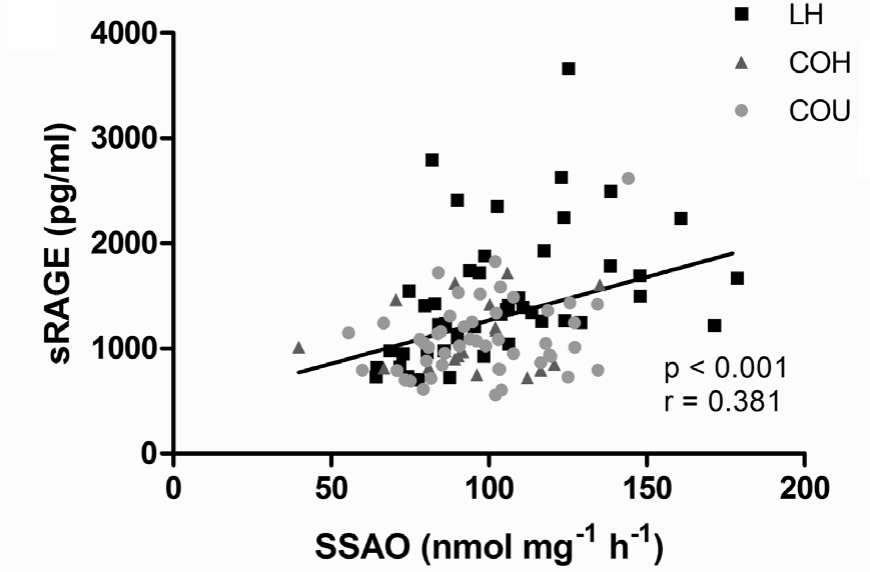
Correlation between activity of semicarbazide-sensitive amine oxidase and soluble receptor for advanced glycation end products levels in lean healthy, centrally obese healthy and unhealthy women. SSAO: activity of semicarbazide-sensitive amine oxidase; sRAGE: soluble receptor for advanced glycation end products; LH: lean healthy; COH: centrally obese healthy; COU: centrally obese unhealthy women.

**Figure 4:**
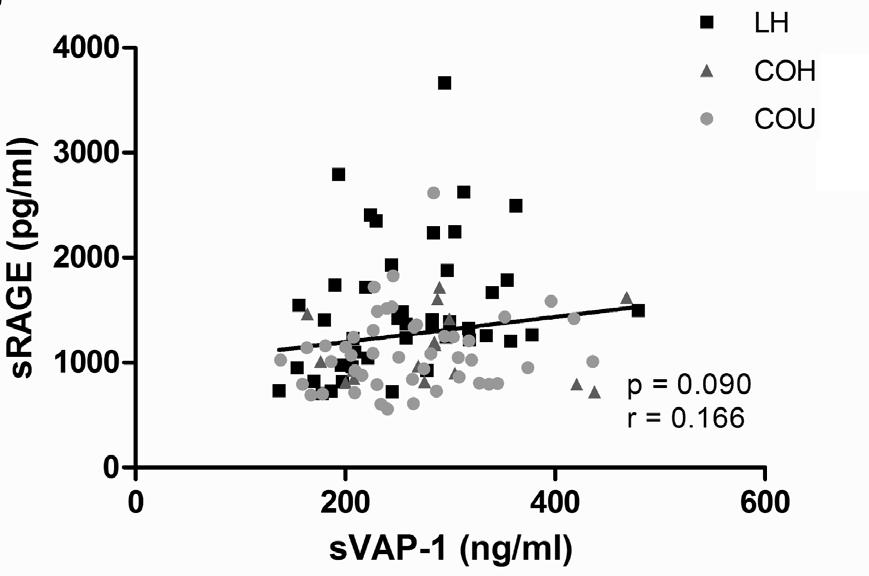
Correlation between concentration of soluble vascular adhesion protein-1 and soluble receptor for advanced glycation end products levels in lean healthy, centrally obese healthy and unhealthy women. SSAO: activity of semicarbazide-sensitive amine oxidase; sRAGE: soluble receptor for advanced glycation end products; LH: lean healthy; COH: centrally obese healthy; COU: centrally obese unhealthy women.

sRAGE levels correlated significantly and inversely with body weight, waist circumference, BMI, WHtR, SBP, DBP, FPG, FPI, LDL-C, VLDL-C, TAG, AIP, uric acid, eGFR, platelet count, hsCRP, and leptin (Table 2). Significant positive relationship of sRAGE with insulin sensitivity, creatinine, adiponectin, and AGE-Fl/Alb has been revealed (Table 2). sRAGE showed a significant positive relationship to SSAO activity (Figure 3), while relationship between sRAGE and sVAP-1 was insignificant (Figure 4). GLM selected WHtR (*P* < 0.001; β: -3234; SE:1023), platelet count (*P* = 0.018; β: -1.60; SE: 0.67), and SSAO activity (*P* = 0.011; β: 6.62; SE: 2.56) as independent significant predictors of sRAGE levels (overall model *P* < 0.001; R^2^: 0.31).

**Table 2:** Pearson or Spearman’s correlation coefficients between concentration of soluble receptor for advanced glycation end products (sRAGE) and anthropometric, clinical, and laboratory variables in women included in the study *

	R	*P*
Weight	-0.357	<0.001
Waist	-0.439	<0.001
BMI	-0.438	<0.001
Waist/height	-0.471	<0.001
SBP	-0.206	0.029
DBP	-0.235	0.013
FPG	-0.280	0.003
FPI	-0.321	0.001
QUICKI	0.340	<0.001
LDL-C	-0.195	0.040
VLDL-C	-0.239	0.012
TAG	-0.213	0.024
AIP	-0.204	0.031
Uric acid	-0.242	0.010
Creatinine	0.245	0.009
eGFR	-0.230	0.015
Plt	-0.287	0.002
hsCRP	-0.348	<0.001
Adiponectin	0.199	0.035
Leptin	-0.308	0.001
SSAO	0.381	<0.001
AGE/Alb	0.197	0.037

*Abbreviations – BMI: body mass index; SBP: systolic blood pressure; DBP: diastolic blood pressure; QUICKI: quantitative insulin sensitivity check index; HDL-C: high density lipoprotein cholesterol; LDL-C: low density lipoprotein cholesterol; VLDL-C: very low density lipoprotein cholesterol; TAG: triacylglycerols; AIP: atherogenic index of plasma; eGFR: estimated glomerular filtration rate (abbreviated MDRD formula); WBC: leukocyte count; RBC: erythrocyte count; Plt: platelet count; hsCRP: high sensitive C-reactive protein; SSAO: activity of semicarbazide-sensitive amine oxidase; sVAP-1: soluble vascular adhesion protein-1; AOPP/Alb: advanced oxidation protein products corrected for plasma albumin; AGE-Fl/Alb: advanced glycation end products-associated fluorescence of plasma corrected for plasma albumin; *italics: Spearman's correlation coefficient;* relationship between sRAGE and cholesterol or HDL-C concentrations, WBC or RBC count, and AGE-Fl/Alb were not significant

### SSAO activity and sVAP-1

**Figure 5:**
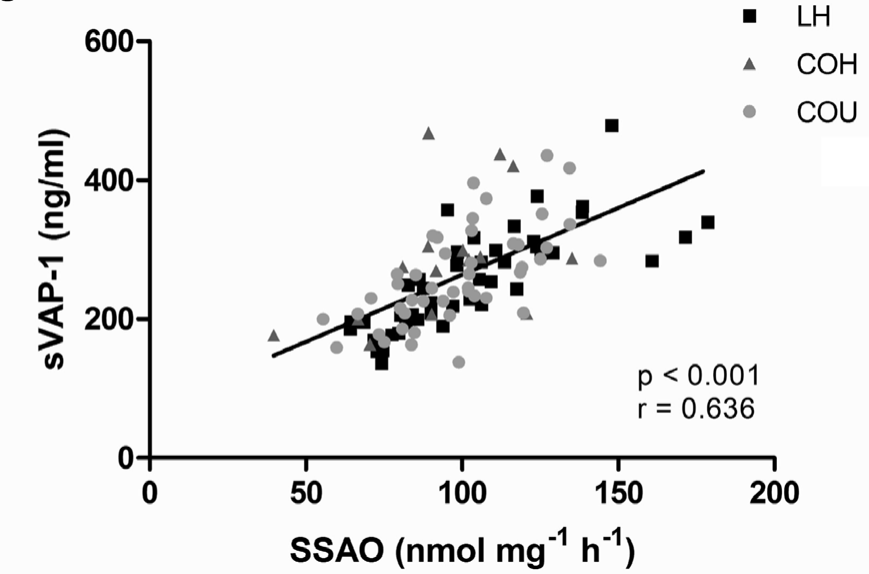
Correlation between activity of semicarbazide-sensitive amine oxidase and soluble vascular adhesion protein-1 levels in lean healthy, centrally obese healthy and unhealthy women. SSAO: activity of semicarbazide-sensitive amine oxidase; sVAP-1: soluble vascular adhesion protein-1; LH: lean healthy; COH: centrally obese healthy; COU: centrally obese unhealthy women.

Except for significant positive relationship with sRAGE (Figure 3), SSAO activity correlated positively with sVAP-1 levels (Figure 5). It also showed significant inverse relationship with BMI (r = -0.190, *P* = 0.047), WHtR (r = -0.214, *P* = 0.025), and ln hsCRP (r = -0.235, *P* = 0.014). GLM selected only sRAGE (*P* < 0.001; β: 0.017; SE: 0.005) as independent significant predictor of SSAO activity (overall model *P* < 0.001; R^2^: 0.14). Except for SSAO activity, sVAP-1 correlated significantly only with erythrocytes count (r = 0.224, *P* = 0.021).

## Discussion

To the best of our knowledge this is the first study investigating sRAGE levels, sVAP-1 and SSAO activity in lean vs metabolically healthy and unhealthy CO women. In contrast to our hypothesis, we revealed similar decline in sRAGE levels in COH and COU women. Moreover, our data show that central obesity is not associated with significant changes in SSAO activity/sVAP-1 levels, regardless of presence or absence of cardiovascular risk factors.

Obesity represents a key risk factor for development of metabolic syndrome, type 2 diabetes, cardiovascular diseases, and certain types of cancer - all of which may lead to increased mortality. However, 10%-to-30% of obese individuals present phenotype free from metabolic abnormalities (12). Thus, the proportion of COH women in our study (18%) corresponded with percentages reported for other populations (12). Metabolically healthy obesity (MHO) does not appear to be a benign condition: the prognosis in MHO individuals for cardiovascular disease is as poor as that in the metabolically unhealthy obese (MUO) (20). Although MHO phenotype carries less risk for type 2 diabetes when compared with MUO, the risk is greater than that in the metabolically healthy normal weight subjects (20,21). In contrast to MUO subjects MHO do not improve significantly their cardiometabolic risk upon weight loss interventions (12).

MHO phenotype is generally defined as presence of obesity (BMI<30.0 kg/m^2^, not taking into account fat distribution), accompanied with <3 cardiometabolic risk factors - elevated BP, TAG, and FPG levels, and decreased HDL-cholesterol (12). However, central obesity, particularly if measured as WHtR, is a better prognostic marker of cardiometabolic risk than BMI (22). Similarly, AIP is a better predictor of atherogenic risk than TAG and HDL-C levels evaluated separately (17). FPG might be maintained within the reference range on the account of hyperinsulinemia, thus FPG is not a reliable proxy of normal glucose homeostasis (12). Thus, we used strict criteria to define MHO phenotype, eg, centrally obese insulin sensitive subject not presenting increased atherogenic risk and elevated BP. We are not aware of any study using zero cardiometabolic risk components to define MHO, and ≤1 additional criteria to obesity are also seldom used in comparison with 2 criteria (23). We showed that even under our strict classification COH women present lower insulin sensitivity, elevated hsCRP and leptin levels if compared with their lean healthy counterparts. MHO was also associated with higher total cholesterol levels, however, only tendency toward unfavorable lipid profile and AIP was observed. Moreover, COH women tended to present higher BP values, uric acid and AOPP/Alb levels, leukocyte and platelet counts if compared with lean healthy subjects – variables which were significantly higher in COU women. Concurrent rise in hsCRP, leukocyte counts and AOPPs (myeloperoxidase reaction-derived products reflecting enhanced activation of phagocytes) (24) points to activation of microinflammation processes. On the other hand, COH women maintained their adiponectin levels despite marked hyperleptinemia – phenomenon reported previously, and potentially contributing to maintenance of insulin sensitivity in MHO phenotype (25).

SRAGE levels are similarly decreased in metabolically healthy and metabolically unhealthy centrally obese women. Although majority of studies in subjects without diabetes (including pre-pubertal children and elderly subjects) report inverse relationship between measures of obesity and sRAGE levels (4,6-9), the data are equivocal (10,11). We did not confirm our hypothesis that this discrepancy might be on the account of MHO phenotype: COH and COU women who did not differ with regard to their measures of obesity displayed similar decline in sRAGE levels. Mechanisms leading to obesity-associated sRAGE decline remain unclear. In homeostasis tissue expression of RAGE is low, and increases with accumulation RAGE ligands (1). In obesity enhanced oxidative stress, microinflammation, and accumulation of lipophilic AGEs induce increased RAGE expression in adipose tissue (26). Thus, if sRAGE levels mirror tissue RAGE expression, plasma sRAGE should be increased in obesity. In obese pre-pubertal children and adolescents esRAGE-to-sRAGE ratio remains constant (27,28). Thus, endogenous secretory and metalloproteinases-shaded isoforms of sRAGE decline proportionally. While no data on ADAM10 expression or activity in obesity are available, plasma MMP-9 levels and its expression in fat tissue of obese subjects are increased (29,30). However, complex interaction of MMPs with their tissue inhibitors does not allow us to speculate whether shading of RAGE is diminished in obesity. Assumption that sRAGE may act as decoy trapping circulating AGEs seems unsubstantiated, as the levels of sRAGE are 3-orders of magnitude lower than even the concentrations of the most abundant plasma AGE – N^ϵ^-(carboxymethyl)lysine (CML) (31). Moreover, plasma CML-to-sRAGE ratio remains constant with increasing number of metabolic syndrome risk factors (4). Circulating levels of hydroimidazolones which bind to RAGE with the highest affinity and specificity among all chemically defined AGEs (15) are not increased in obesity (32). However, another RAGE ligand – pro-inflammatory cytokine high mobility group protein box-1 (HMGB1), displaying 10-fold higher binding affinity to RAGE in comparison with AGEs, circulates in concentrations similar to those of sRAGE, and has been shown to be increased in obesity (33). HMGB1 is secreted by preadipocytes and controls inflammation (ie, the secretion of interleukine-6 and monocyte chemotactic protein-1) in adipose tissues through the binding to RAGE (34). In general population sRAGE independently and inversely associated with HMGB1 (35). Herein we showed negative relationship between inflammatory markers (hsCRP, AOPP/Alb) and sRAGE. Whether decreased sRAGE level in obesity reflects mainly the pro-inflammatory status, particularly associated with high HMGB1 levels, remains to be confirmed. In large community-based population study in subjects without diabetes low levels of sRAGE were significantly associated with future risk of diabetes, coronary heart disease, and mortality (36). In healthy adult women lower sRAGE levels reflected accumulation of the epicardial visceral fat (9). Thus, low sRAGE levels associating with microinflammatory status suggest that despite an absence of standard cardiometabolic risk factors MHO women might be on increased risk to develop cardiometabolic disease in future. As sRAGE concentrations remain relatively stable over years, it has been suggested that a single measure of circulating sRAGE may be sufficient for characterizing long-term risk in the general population (37).

SSAO/sVAP-1 are not altered in metabolically healthy and unhealthy centrally obese women. VAP-1 represents a molecule with a dual action: as adhesion molecule it favors lymphocyte recruitment into site of inflammation; possessing enzyme activity of semicarbazide-sensitive amine oxidase (SSAO, EC 1.4.3.21) it converts primary amines into corresponding aldehydes (eg, aminoacetone into methylglyoxal), generating H_2_O_2_ and ammonia (14). Thus, as expected, sVAP-1 and plasma SSAO activity showed tight correlation in our study. The membrane form of SSAO/VAP-1, highly expressed in endothelial cells, regulates leukocyte trafficking into site of inflammation (38). In obesity the membrane-bound VAP-1 is abundantly expressed on white fat cells, and is supposed to contribute to adipogenesis and pathological energy metabolism (38). It has been suggested that in obesity VAP-1 remains in the membrane-bound form, with a concurrent decrease in the circulating sVAP-1 concentration (39). On the other hand, a different study reported an elevated plasma SSAO activity in morbidly obese patients (40); while in our study neither concentrations of sVAP-1, nor the plasma SSAO activity differed significantly between lean and centrally obese women, regardless of their phenotype. Clinical data on sVAP-1/SSAO in obesity are scares, with contradictory results, and require further aimed investigations.

Formation of AGEs is accelerated under conditions of enhanced oxidative stress such as obesity, and methylglyoxal is considered to be the most potent glycation agent (26). Both, enhanced oxidative stress and methylglyoxal may induce insulin resistance (41). Similarly to RAGE, VAP-1 is shaded by MMPs (13). Thus, we assumed that in obesity sRAGE, sVAP-1 levels and SSAO activity might associate. However, in contrast to sRAGE levels SSAO activity and sVAP-1 were not altered in centrally obese women. Obese individuals present approximately 35% higher plasma methylglyoxal levels in comparison with their lean counterparts (32), but circulating methylglyoxal levels show no significant relationship with measures of obesity (42). Our data suggest that obesity-associated rise in methylglyoxal is not due to increase in SSAO activity. Despite its unchanged activity in obesity, SSAO showed positive relationship with sRAGE, and in multivariate analyses appeared as a significant independent predictor to sRAGE levels. Equivocal results of statistical analyses do not allow any speculation on potential interplay of RAGE/SSAO/VAP-1 systems in central obesity – further studies are definitely needed.

The strengths of our study include recruitment of White Caucasian women of Middle European descent, without diabetes, of similar age, from geographically restricted area of South-Western Slovakia; strict criteria used to define MHO; determination of variety of non-standard cardiometabolic risk markers; and complex statistical approach. The weaknesses of this study are largely related to its cross-sectional design, not allowing elucidation of the causal relationships between circulating sRAGE, sVAP-1, SSAO activity and anthropometric, metabolic, and inflammatory variables, ie, issues better examined in a longitudinal study.

### Conclusion

Our data suggest that by strict criteria classified centrally obese women present abnormalities in non-standard markers of cardiometabolic risk, emphasizing that there is no healthy pattern of obesity. The clinical impact of low sRAGE levels for future prognosis of metabolically healthy obese subjects, and potential interaction in RAGE/VAP-1/SSAO axis in obesity-associated pathology remain to be elucidated in longitudinal studies.
